# α‐Synuclein toxicity in yeast and human cells is caused by cell cycle re‐entry and autophagy degradation of ribonucleotide reductase 1

**DOI:** 10.1111/acel.12922

**Published:** 2019-04-11

**Authors:** Belém Sampaio‐Marques, Ana Guedes, Igor Vasilevskiy, Susana Gonçalves, Tiago F. Outeiro, Joris Winderickx, William C. Burhans, Paula Ludovico

**Affiliations:** ^1^ School of Medicine, Life and Health Sciences Research Institute (ICVS) University of Minho Braga Portugal; ^2^ ICVS/3B’s ‐ PT Government Associate Laboratory Guimarães Portugal; ^3^ Faculdade de Ciências Médicas, CEDOC – Chronic Diseases Research Center Universidade Nova de Lisboa Lisboa Portugal; ^4^ Department of Experimental Neurodegeneration, Center for Nanoscale Microscopy and Molecular Physiology of the Brain (CNMPB) University Medical Center Göttingen Göttingen Germany; ^5^ Center for Biostructural Imaging of Neurodegeneration Göttingen Germany; ^6^ Max Planck Institute for Experimental Medicine Göttingen Germany; ^7^ Functional Biology KU Leuven Heverlee Belgium; ^8^ Department of Molecular and Cellular Biology Roswell Park Cancer Institute Buffalo New York

**Keywords:** alpha‐synuclein, autophagy, cell cycle re‐entry, chronological aging, DNA damage responses, ribonuclease reductase

## Abstract

α‐Synuclein (aSyn) toxicity is associated with cell cycle alterations, activation of DNA damage responses (DDR), and deregulation of autophagy. However, the relationships between these phenomena remain largely unknown. Here, we demonstrate that in a yeast model of aSyn toxicity and aging, aSyn expression induces Ras2‐dependent growth signaling, cell cycle re‐entry, DDR activation, autophagy, and autophagic degradation of ribonucleotide reductase 1 (Rnr1), a protein required for the activity of ribonucleotide reductase and dNTP synthesis. These events lead to cell death and aging, which are abrogated by deleting *RAS2*, inhibiting DDR or autophagy, or overexpressing *RNR1*. aSyn expression in human H4 neuroglioma cells also induces cell cycle re‐entry and S‐phase arrest, autophagy, and degradation of RRM1, the human homologue of *RNR1*, and inhibiting autophagic degradation of RRM1 rescues cells from cell death. Our findings represent a model for aSyn toxicity that has important implications for understanding synucleinopathies and other age‐related neurodegenerative diseases.

## 1 INTRODUCTION

α‐Synuclein (aSyn) is a presynaptic neuronal protein that is genetically and neuropathologically linked to a group of age‐related neurodegenerative diseases called synucleinopathies. The abnormal accumulation of aSyn aggregates is characteristic of this group of disorders that includes, among others, Parkinson's disease (PD). aSyn has been implicated in diverse physiological processes including the regulation of calcium and mitochondrial homeostasis, polyunsaturated fatty acids levels, and chaperone activity (Wales, Pinho, Lazaro, & Outeiro, 2013). However, the normal physiological functions of aSyn as well as the nature of the cytopathic effects of aSyn overexpression and mutations remain incompletely understood. An emerging body of evidence suggests that cell cycle aberrations, including inappropriate re‐entry into the cell cycle, are linked to the pathological effects of aSyn (Sharma et al., 2017; Tokarz, Kaarniranta, & Blasiak, 2016). For example, elevated levels of proteins involved in cell cycle re‐entry and DNA synthesis in dopaminergic neurons are observed in the brain of PD patients (Hoglinger et al., 2007). Cell cycle re‐entry, DNA damage, activation of DNA damage responses (DDR), and cell death have also been observed in numerous cellular and animal models of PD and other synucleinopathies (Camins et al., 2010; Lee et al., 2003; Paiva et al., 2017; Smith et al., 2003). Furthermore, inhibition of cyclin‐dependent kinases (Cdks) can induce neuroprotective effects (Rideout, Wang, Park, & Stefanis, 2003). Together, evidence from studies of humans as well as from experimental models strongly suggests a role for cell cycle re‐entry and DDR in the mechanisms of aSyn pathobiology.

Mounting evidence suggests that macroautophagy (hereafter autophagy), a self‐degradation pathway, plays a key role in the DDR by controlling the levels of proteins involved in cell cycle checkpoints and DNA synthesis/repair mechanisms. Particularly relevant is the discovery that in the model organism *Saccharomyces cerevisiae* (budding yeast), DNA damage induces the autophagic degradation of ribonucleotide reductase 1 (Rnr1) (Dyavaiah, Rooney, Chittur, Lin, & Begley, 2011), which is the large subunit of ribonucleotide reductase (RNR), a highly conserved enzyme that catalyzes the formation of deoxyribonucleotides required for both DNA replication and repair. In budding yeast, DDR was also found to activate a selective pathway of autophagy, termed genotoxin‐induced targeted autophagy (GTA), which requires the involvement of the Mec1 and Rad53 kinases, as well as a central component of the selective autophagy machinery, Atg11 (Eapen et al., 2017). Also relevant here is the recent discovery that Mec1 plays a fundamentally important role in protein homeostasis (Corcoles‐Saez et al., 2018).

Budding yeast has been extensively employed in models of PD and other synucleinopathies (Tenreiro, Franssens, Winderickx, & Outeiro, 2017). Previously, we showed in the budding yeast chronological aging model that aSyn toxicity is associated with the enhanced autophagy that depends on Atg11 (Sampaio‐Marques et al., 2012). Here, we show that in quiescent stationary‐phase budding yeast cells, which mimic the quiescent state of postmitotic neurons, aSyn expression promotes cell cycle re‐entry, S‐phase arrest, and DDR activation. The induction of DDR is responsible for a dramatic increase in autophagy, which in turn causes the degradation of Rnr1 and cell death that leads to premature aging in the budding yeast chronological aging model. Expression of aSyn in human H4 neuroglioma cells also induces the accumulation of cells in S‐phase, autophagy and the degradation of RRM1, the human homologue of Rnr1, and cell death, which is blocked by inhibiting autophagy. These findings reveal a novel mechanism for aSyn toxicity in aged postmitotic cells that involves the inappropriate entry of cells into S‐phase followed by DDR and the autophagy‐dependent loss of RNR activity.

## 2 RESULTS

### 2.1 aSyn toxicity in budding yeast cells is associated with cell cycle re‐entry, S‐phase arrest, and increased autophagy

aSyn promotes autophagy and mitochondrial dysfunction; however, the relationship between metabolic stress, autophagy, DNA damage responses (DDR), and cell death induced by aSyn remains poorly understood. To learn more about these phenomena and how they might be related to re‐entry of quiescent cells into the cell cycle, wt aSyn (aSyn) or the PD‐associated mutant A30P aSyn, which is not toxic in budding yeast cells (Outeiro & Lindquist, 2003), was constitutively expressed in wild‐type yeast cells.

**Figure 1:**
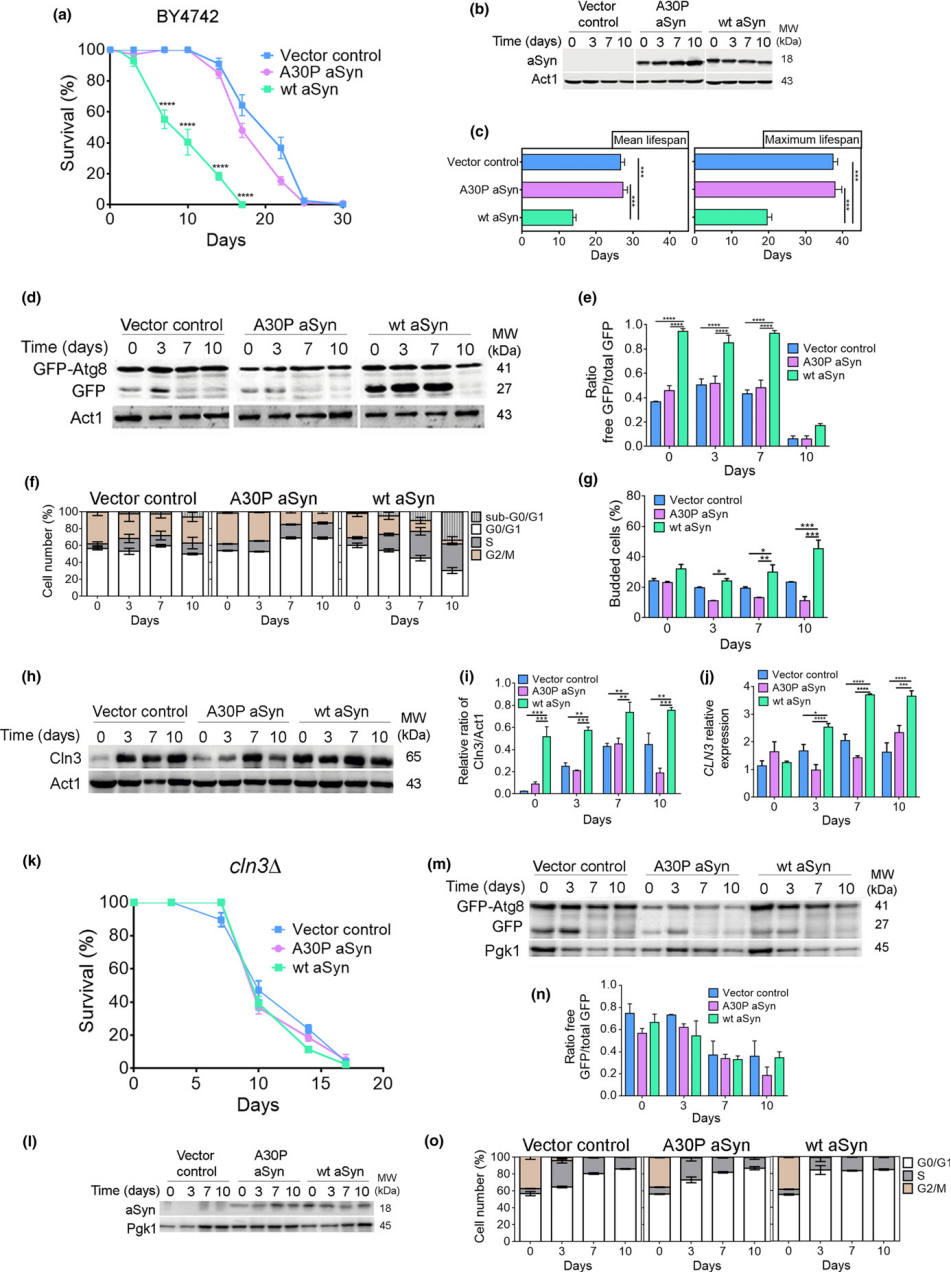
aSyn promotes cell cycle re‐entry and S‐phase arrest associated with increased autophagy. (a) Chronological lifespan (CLS), (b) aSyn levels, and (c) mean lifespan and maximum lifespan of BY4742 cells expressing the vector control, wt aSyn, or the A30P aSyn variant. (d) Representative blot and (e) graphical representation of the GFP‐Atg8 processing assay. (f) Cell cycle analysis. (g) Bud index. (h) Representative blot of Cln3. (i) Graphical representation of the Cln3/Act1. (j) Relative *CLN3* mRNA levels of BY4742 cells expressing the vector control or aSyn variants. (k) CLS, (l) aSyn levels, (m) representative blot, and (n) graphical representation of the GFP‐Atg8 processing assay for *cln3Δ* cells expressing the vector control or aSyn variants. (o) Cell cycle analysis. Significance of the data was determined by two‐way ANOVA (*p ≤ 0.05, **p ≤ 0.01; ***p ≤ 0.001, ****p ≤ 0.0001) comparing wild‐type or *cln3Δ* cells expressing vector control or the aSyn variants

The heterologous expression of human wild‐type aSyn in budding yeast cells is accompanied by enhanced autophagy and shortening of chronological lifespan (CLS), which was assessed by determining how long cells survive in a quiescent, stationary‐phase state (Figure 1a–e and Supporting information Figure S1) (Sampaio‐Marques et al., 2012). These observations were associated with a time‐dependent increase in the percentage of aSyn‐expressing cells accumulating in S‐phase, suggesting entry of stationary‐phase cells into S‐phase followed by cell cycle arrest, in contrast with the typical G0/G1 cell cycle arrest observed in stationary‐phase cells harboring the vector control or expressing the A30P aSyn nontoxic variant (Figure 1f). Re‐entry of quiescent cells into the cell cycle was also indicated by an increased bud index detected in cells expressing aSyn (Figure 1g). An increased percentage of aSyn‐expressing cells with a DNA content less than G0/G1 (Figure 1f) was also observed, consistent with the previously described aSyn‐induced apoptotic cell death (Flower, Chesnokova, Froelich, Dixon, & Witt, 2005) and with the survival data presented in Figure 1a.

Cyclin Cln3, required for the G1‐to‐S transition, was increased at both the mRNA and protein level in cells expressing aSyn in comparison with cells expressing the nontoxic A30P aSyn variant or the vector control (Figure 1h‐j). Our results also showed that *CLN3* deletion abrogates aSyn toxicity as observed by the overlapped CLS curves associated with an autophagy flux similar to cells expressing the nontoxic A30P aSyn variant or the vector control, and decreased percentage of cells in S‐phase (Figure 1k–o). To support the crucial role of Cln3 in the transition from G1 phase to S‐phase, the expression levels of *CLN1* and *CLN2,*which assist Cln3 in this transition, and of the S‐phase cyclins *CLB5* and *CLB6*, were evaluated. The results revealed a significant increased expression of all of these cyclins in aSyn‐expressing cells during CLS (Supporting information Figure S2). Notably, aSyn toxicity is dependent on the molecular chaperone Ydj1, which mediates the release of Cln3 from the ER, its nuclear accumulation, and cell cycle entry (Verges, Colomina, Gari, Gallego, & Aldea, 2007) (Supporting information Figure S2). These results unequivocally prove that aSyn causes cells to re‐enter the cell cycle. They establish a previously unknown relationship between aSyn toxicity, enhanced autophagy, cell cycle re‐entry, and S‐phase arrest in aged budding yeast cells, phenomena that are independent of yeast strain background (Supporting information Figure S3).

### 2.2 Abrogation of Ras2 signaling abolishes aSyn toxicity by inducing a G0/G1 arrest, inhibition of DNA damage responses, and autophagy

**Figure 2:**
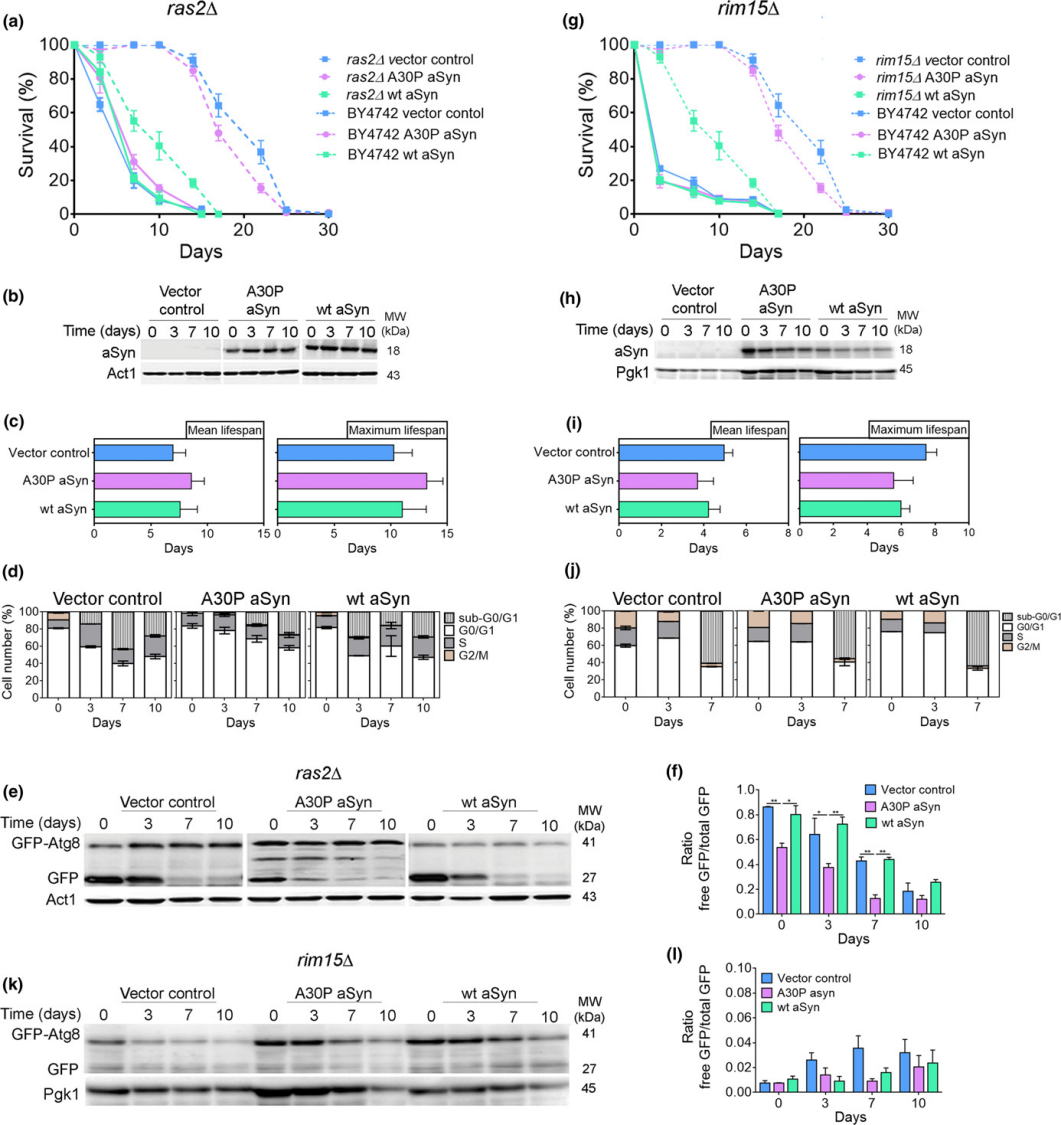
Cell cycle arrest at G0/G1 phase abrogates aSyn toxicity. (a,g) CLS, (b,h) aSyn levels, (c,i) mean lifespan and maximum lifespan, (d,j) cell cycle analysis, (e,k) representative blot, and (f,l) graphical representation of the GFP‐Atg8 processing assay of *ras2Δ* and *rim15Δ* cells expressing the vector control or aSyn variants, respectively (CLS of BY4742, dashed lines, are repeated from Figure 1a to facilitate interpretation). Significance of the data was determined by two‐way ANOVA (*p ≤ 0.05, **p ≤ 0.01) comparing *ras2Δ* or *rim15Δ* cells expressing vector control or the aSyn variants

In budding yeast, abrogation of *RAS2*, a gene encoding a member of a family of small GTPases conserved in humans, is known to induce an efficient G0/G1 arrest in stationary phase that protects cells against DNA replication stress and damage (Weinberger et al., 2007). To determine whether an efficient G0/G1‐phase cell cycle arrest induced in budding yeast cells by abrogating RAS signaling can protect yeast cells from aSyn toxicity, aSyn variants were constitutively expressed in *ras2Δ* cells. *RAS2*deletion abolished aSyn‐negative effects on longevity, resulting in a CLS similar to those obtained in vector control or the A30P aSyn variant‐expressing cells (Figure 2a,b), which was confirmed by the mean lifespan and maximum lifespan (Figure 2c). Compared to wild‐type cells (Figure 1f,g), *ras2Δ* cells expressing aSyn also less frequently entered into or accumulated in S‐phase (Figure 2d). Autophagy decreased to levels observed in control cells (Figure 2e,f) suggesting that enhanced autophagy in aSyn‐expressing cells depends on cell cycle re‐entry. To further support the hypothesis that an efficient arrest in G0/G1 phase abrogates aSyn toxicity, aSyn variants were constitutively expressed in cells deleted of *RIM15*, a serine/threonine kinase required for CLS extension promoted by abrogation of nutrient signaling pathways or caloric restriction. The results showed that aSyn toxicity is abolished in *rim15Δ* cells (Figure 2g–i), and this phenotype is associated with an efficient arrest in G0/G1 phase and decreased autophagy (Figure 2j–l). These results are in agreement with those we previously reported showing that the shorter CLS induced by aSyn is abolished by caloric restriction, a physiological intervention that promotes an efficient arrest of cells in G0/G1 through the maintenance of autophagy at homeostatic levels (Guedes, Ludovico, & Sampaio‐Marques, 2016). Our data establish that the shorter CLS and premature death of cells expressing aSyn are associated with Ras2‐dependent cell cycle re‐entry, S‐phase arrest, and increased autophagy, which are prevented by forcing cells to efficiently arrest in G0/G1 phase.

**Figure 3:**
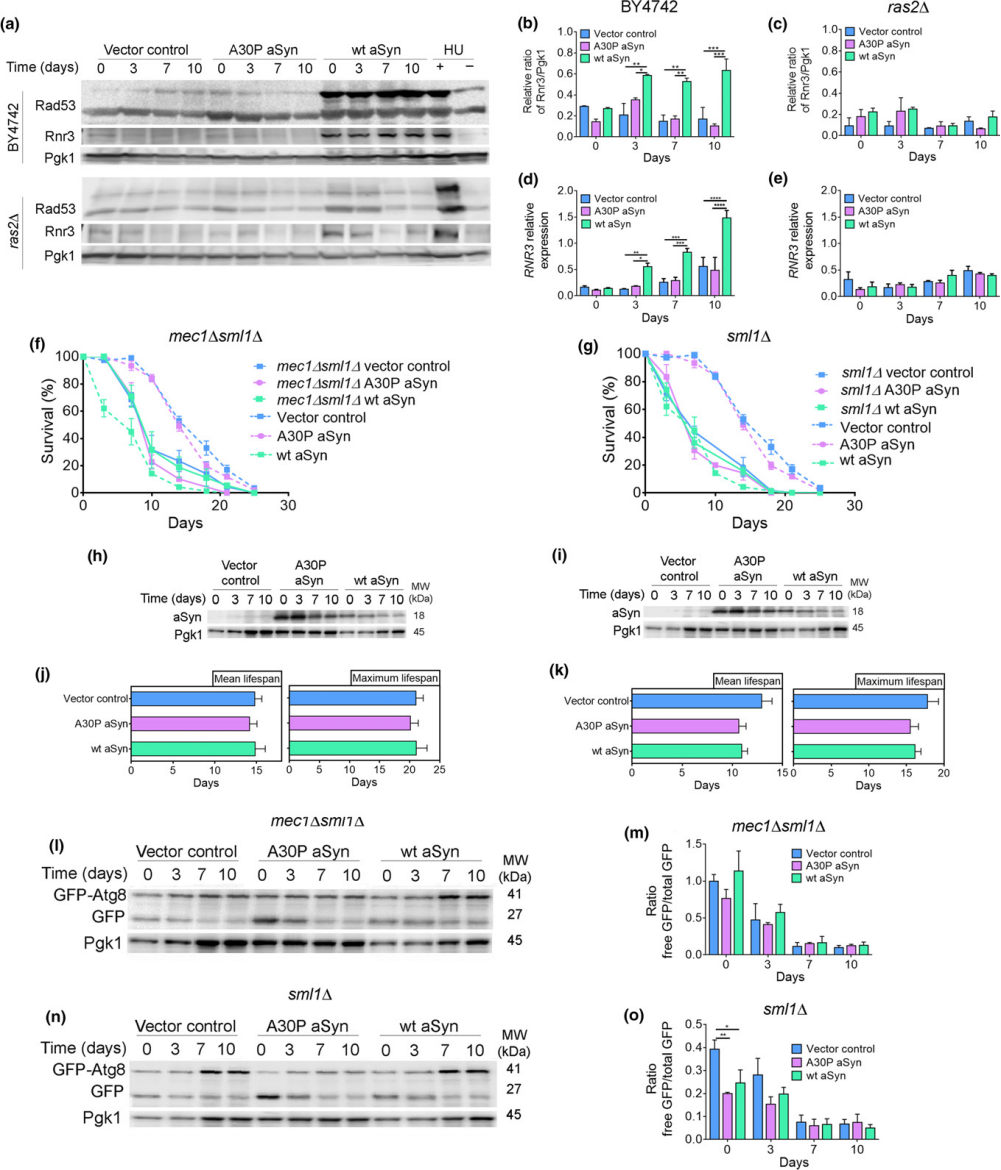
aSyn promotes DNA damage responses. (a) Representative blots for Rad53 phosphorylation and Rnr3 levels. (b,c) Graphical representation of the Rnr3/Pgk1 and (d,e) relative *RNR3*mRNA levels of BY4742 or *ras2Δ* cells expressing the vector control or aSyn variants, respectively. As a positive or negative control of DDR activation, cells treated or not with hydroxyurea (HU) were used. Significance of the data was determined by two‐way ANOVA (*p ≤ 0.05, **p ≤ 0.01, ***p ≤ 0.001, ****p ≤ 0.0001) comparing BY4742 or *ras2Δ* cells expressing vector control or the aSyn variants. (f,g) CLS and (h,i) aSyn levels of *mec1Δsml1Δ* and *sml1Δ* cells, respectively, expressing the vector control or aSyn variants (CLS data from W303‐1A wild‐type cells, dashed lines, are repeated from Supporting information Figure S3 to facilitate interpretation). (j,k) Mean lifespan and maximum lifespan of *mec1Δsml1Δ* and *sml1Δ* cells. (l,n) Representative blots and (m,o) graphical representation of the GFP‐Atg8 processing assay. Significance of the data was determined by two‐way ANOVA (*p ≤ 0.05, **p ≤ 0.01) comparing *mec1Δsml1Δ* or *sml1Δ*cells expressing vector control or aSyn variants

Recently, it was demonstrated that DDR induces a selective autophagy pathway dependent on the Mec1 and Rad53 kinases (Eapen et al., 2017). Cells expressing aSyn, but not cells harboring the vector control or expressing the A30P aSyn variant, exhibited Rad53 mobility shift, and Rnr3 increased levels (both mRNA and protein) (Figure 3a–e) consistent with the activation of the DDR (Hendry, Tan, Ou, Boone, & Brown, 2015). The mobility of Rad53 remained unaltered, and the Rnr3 levels decreased in *ras2Δ* cells (Figure 3a–e). Thus, aSyn toxicity is associated with the induction of DDR, which can be prevented by an efficient arrest in G0/G1 cell cycle phase.

As cells enter S‐phase, inducing DNA damage or by replication stress, the Mec1/Rad53 kinase cascade leads to the phosphorylation of Sml1, targeting it for degradation and activating RNR. *RNR* genes expression is also induced at S‐phase by the Mbp1‐Swi6 complex and by the Mec1/Rad53/Dun1 kinase cascade after DNA damage (Zhao, Chabes, Domkin, Thelander, & Rothstein, 2001). To elucidate the role of DDR on autophagy induction, aSyn was expressed in *mec1Δ sml1Δ* cells and in *sml1Δ* cells. In contrast to its expression in wild‐type cells, expression of aSyn did not alter the CLS of *mec1Δ sml1Δ* cells (Figure 3b,c,f). The same was observed for *sml1Δ* cells expressing aSyn (Figure 3d,e,g). Importantly, compared to wild‐type cells (Supporting information Figure S3), *mec1Δ sml1Δ* and *sml1Δ* cells expressing aSyn displayed reduced autophagy (Figure 3h–k). Therefore, aSyn expression induces Ras2‐dependent cell cycle re‐entry, S‐phase arrest, and DDR activation, which induces a selective autophagy pathway in concert with a shorter CLS.

### 2.3 Autophagy‐dependent degradation of Rnr1 is a proximate cause of aSyn toxicity in budding yeast cells

**Figure 4:**
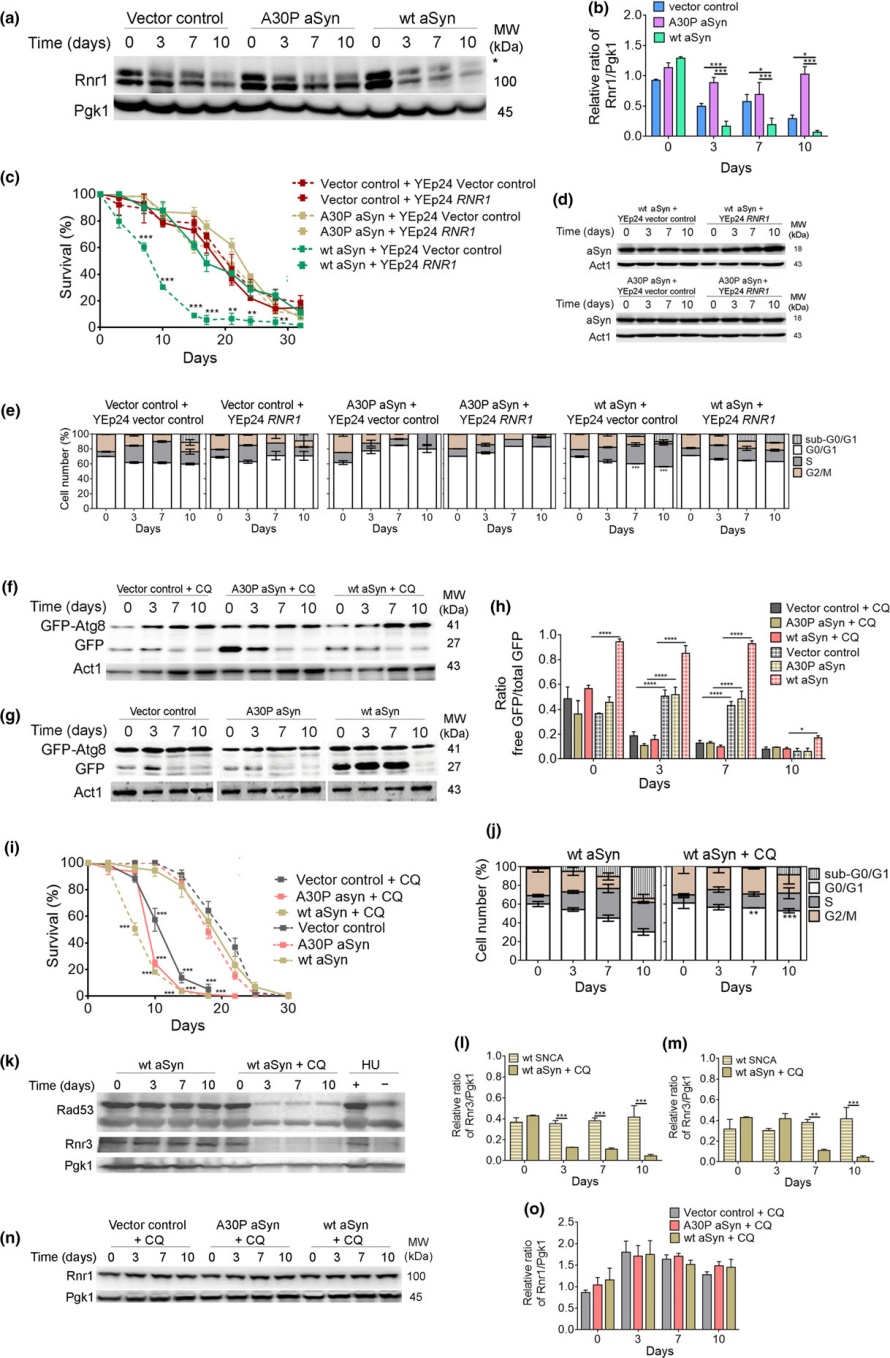
Decreased levels of Rnr1 in aSyn‐expressing cells are restored by autophagy inhibition. (a) Representative blot for Rnr1 of BY4742 cells expressing the vector control or aSyn variants and (b) graphical representation of the Rnr1/Pgk1. *indicates a nonspecific band in the anti‐Rnr1 immunoblot. Significance of the data was determined by two‐way ANOVA (*p ≤ 0.05, ***p ≤ 0.001) comparing wild‐type cells expressing the vector control or the aSyn variants. (c) CLS, (d) aSyn levels, and (e) cell cycle analysis of BY4742 cells co‐expressing the vector control or aSyn variants and *RNR1* or vector control. Significance of the data was determined by two‐way ANOVA (**p ≤ 0.01; ***p ≤ 0.001) comparing cells co‐expressing *RNR1* and the vector control or aSyn variants. (f,g) Representative blots of the GFP‐Atg8 processing assay of BY4742 cells expressing the vector control or aSyn variants in the presence or absence of chloroquine (CQ) (50 µg/µl added at day 0 of CLS). (h) Graphical representation of the GFP‐Atg8 processing assay. (i) CLS of BY4742 cells expressing the vector control or aSyn variants in the presence (solid lines) or absence (dashed lines) of CQ. (j) Cell cycle analysis of BY4742 cells expressing wt aSyn treated or not with CQ. (Data on Figure 4g,I,j for BY4742 cells expressing the vector control or aSyn variants without CQ are repeated from Figure 1 to facilitate interpretation). Significance of the data from h–j was determined by two‐way ANOVA (*p ≤ 0.05, **p ≤ 0.01; ***p ≤ 0.001, ****p ≤ 0.0001) comparing BY4742 cells expressing the vector control or aSyn variants in the presence or absence of CQ. (k) Representative blots for Rad53 phosphorylation and Rnr3. (l) Graphical representation of the Rnr3/Pgk1 and (m) relative *RNR3*mRNA levels of BY4742 cells expressing wt aSyn in the absence or presence of CQ. As a positive or negative control for DDR activation, extracts from cells treated with hydroxyurea (HU) were used. Significance of the data was determined by Student's *t* test (**p ≤ 0.01; ***p ≤ 0.001) comparing BY4742 cells expressing the vector control or aSyn variants in the presence or absence of CQ. (n) Representative blot for Rnr1 and (o) graphical representation of the Rnr1/Pgk1 of BY4742 cells expressing the vector control or aSyn variants treated with CQ

In budding yeast, ribonucleotide reductase 1 (Rnr1), which is the large subunit of RNR, an enzyme that maintains dNTP pools required for DNA synthesis and repair, is degraded by autophagy (Dyavaiah et al., 2011). We next asked whether S‐phase arrest in cells expressing aSyn might be related to induction of autophagy by DDR followed by degradation of Rnr1. Enhanced autophagy observed after expression of aSyn (Figure 1d,e) coincided with a substantial reduction in levels of Rnr1 in stationary‐phase cells (Figure 4a,b). The increased levels of Rnr1 in A30P aSyn‐expressing cells (Figure 4a,b) are, most probably, explained by a reduced degradation of Rnr1 in these cells in concert with an increased number of A30P‐containing vesicles (Flower et al., 2007).

*RNR1*overexpression abolished aSyn toxicity as indicated by an increase in CLS (Figure 4c,d and Supporting information Figure S4). *RNR1* overexpression also enhanced G0/G1‐phase arrest (Figure 4e). Therefore, the reduction in levels of Rnr1 contributes to the toxic effects of aSyn, most likely by trapping cells in S‐phase and reducing pools of dNTPs below levels required for efficient DNA replication. To determine whether the reduction in levels of Rnr1 is caused by DDR‐dependent autophagy, autophagy was pharmacologically inhibited by chloroquine (CQ), which increases vacuolar pH and inhibits vacuole–autophagosome fusion (Lenz & Holzer, 1984). As we previously reported (Sampaio‐Marques et al., 2012), autophagy inhibition by CQ (Figure 4f–h) abolished aSyn toxicity indicated by an extension of CLS in aSyn‐expressing cells (Figure 4i and Supporting information Figure S4). Furthermore, when autophagy was inhibited, a lower percentage of cells arrested in S‐phase was observed (Figure 4j and Supporting information Figure S4) and the Rad53 mobility pattern and Rnr3 levels were similar to control cells (Figure 4k,l). Importantly, autophagy inhibition restored Rnr1 levels (Figure 4l). All of these findings point to autophagy‐dependent Rnr1 destruction downstream of aberrant cell cycle re‐entry and DDR as a proximate cause of aSyn‐induced cell death.

### 2.4 aSyn expression in human cells promotes the same toxicity‐associated phenotypes observed in budding yeast cells

**Figure 5:**
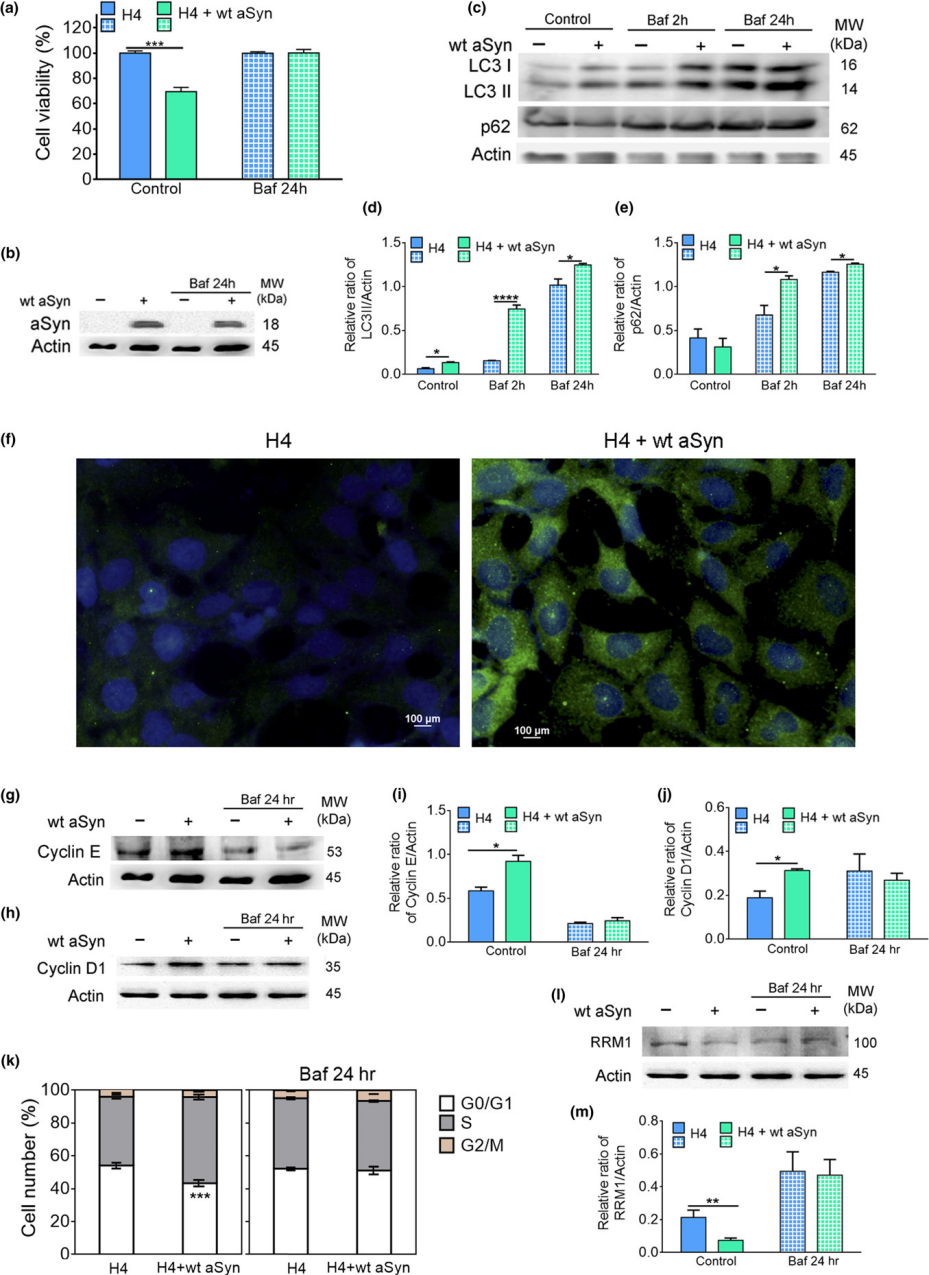
aSyn promotes S‐phase cell cycle arrest, increases autophagy, and decreases RRM1 levels in human cells. (a) Cellular viability and (b) aSyn levels of H4 cells transiently transfected with the vector control or wt aSyn in the absence (control) or presence of bafilomycin A1 (Baf) (10 nM for 24 hr). (c) Representative blot of LC3 processing and p62 for autophagy flux assessment. (d,e) Graphical representation of the LC3II/actin and p62/actin, respectively. For the evaluation of autophagy flux, control samples expressing the vector control or wt aSyn were incubated for 2 hr with Baf (10 nM). (f) Representative image of microscopy visualization of LC3 puncta. Cells were stained with an antibody against anti‐LC3 (green) and counterstained with DAPI to label nuclei (blue). Bar = 100 µm. (g,h) Representative blot of cyclin E and D1, respectively. Graphical representation of (i) cyclin E and (j) cyclin D1 protein levels. (k) Cell cycle analysis. (I) Representative blot of RRM1 levels and (m) graphical representation of the RRM1/actin. Significance of data was determined by Student's *t* test (*p ≤ 0.05, **p ≤ 0.01, ***p ≤ 0.001, ****p ≤ 0.0001) between H4 cells expressing the vector control or wt aSyn untreated or treated with Baf for 2 hr or 24 hr

To extend our observations to cultured human neuronal cells, we transiently transfected human H4 neuroglioma cells with aSyn or vector control. The involvement of autophagy in aSyn toxicity (Figure 5a,b) was assessed by analyzing LC3 (mammalian homologue of yeast Atg8) processing and p62 by immunoblotting and by LC3 puncta visualization in the presence and absence of lysosomal degradation, promoted by treatment for 2 hr with bafilomycin A1, a specific inhibitor of the vacuolar ATPase that inhibits the acidification of lysosomes (Klionsky et al., 2016). Inhibition of autophagy for 2 hr with bafilomycin A1 resulted in an increase of LC3‐II and p62 levels (Figure 5c–e) and LC3 puncta (Figure 5f) in cells expressing aSyn indicating an enhancement of autophagy flux, as we observed in budding yeast cells. Similar to budding yeast, inhibition of autophagy during aSyn expression by treatment for 24 hr with bafilomycin A1 resulted in increased cell viability (Figure 5a–e). Expression of aSyn also increased the levels of cyclin E and D1, which are required for the G1‐to‐S transition in human cells (Figure 5g–j), as well as the number of cells accumulating in S‐phase (Figure 5k).

Immunoblot analysis of RRM1 indicated that its levels were decreased in response to aSyn expression (Figure 5l,m). Inhibition of autophagy during aSyn expression (24 hr) restored RRM1 levels and abrogated aSyn toxicity. Thus, similar to budding yeast, aSyn expression in H4 cells is toxic and causes cell cycle re‐entry and arrest in S‐phase accompanied by increased autophagy and an autophagy‐dependent reduction in levels of RRM1. Furthermore, the reduced levels of RRM1 are likely a proximate cause of aSyn toxicity (Figure 5).

## 3 DISCUSSION

Inappropriate re‐entry into the cell cycle of postmitotic neurons followed by the death of these cells is frequently observed in PD and other synucleinopathies (Folch et al., 2012; Lee et al., 2003; Lombardi & Lasagni, 2016; Paiva et al., 2017; Sharma et al., 2017; Smith et al., 2003). Furthermore, deregulation of cellular proteolytic systems, particularly autophagy, also appears to play an important role in synucleinopathies (Sampaio‐Marques & Ludovico, 2015; Xilouri, Brekk, & Stefanis, 2013). However, how these phenotypes relate to each other and how they might contribute to aSyn toxicity have remained poorly understood.

Here, we show both in the budding yeast chronological aging model and in human neuroglioma cells that expression of aSyn induces inappropriate cell cycle re‐entry of quiescent postmitotic cells in the absence of the nutrient signaling in budding yeast or growth factor signaling in H4 neuroglioma cells that normally drive entry of these cells into the cell cycle. Cell cycle re‐entry of postmitotic neurons in synucleinopathies and other neurodegenerative disorders, which is frequently assessed by bromodeoxyuridine incorporation as these cells enter S‐phase, is a generally, but not universally, accepted phenotype, because it can be difficult to distinguish small amounts of DNA replication from DNA repair. In our budding yeast experiments, cell cycle re‐entry induced by aSyn expression was also revealed by more frequent budding of cells, indicating that aSyn induction of cell cycle re‐entry in postmitotic cells can be detected independently of measurements of DNA content. To our knowledge, our data provide the first evidence that the aSyn‐induced re‐entry into the cell cycle detected in PD and other synucleinopathies can be recapitulated in the model organism budding yeast. This should facilitate the molecular dissection of this specific phenotype using the powerful genetics and other tools available in the model organism budding yeast.

In addition, inhibition of cell cycle re‐entry of budding yeast cells expressing aSyn by forcing these cells to arrest the cell cycle at G0/G1 phase by *RAS2* or *RIM15* deletion (Figure 2) or caloric restriction (Guedes et al., 2016), as well as abrogation of DDR by genetic ablation of the *MEC1* kinase, protects against aSyn‐mediated toxicity, resulting in an extended CLS. Interestingly, aSyn expression in rat PC12 cells also induces RAS signaling in concert with the accumulation of cells in S‐phase (Lee et al., 2003), which suggests that growth signaling by RAS‐dependent pathways is a conserved feature of this phenotype of synucleinopathies. Together, these findings support an active role of RAS‐dependent cell cycle re‐entry and DDR in promoting the toxic effects of expressing aSyn in quiescent yeast cells and by extension in postmitotic mammalian neurons. This is consistent with emerging models of the toxic effects of cell cycle re‐entry in synucleinopathies and other neurodegenerative disorders (Sharma et al., 2017; Tokarz et al., 2016). Postmitotic cells may enter the cell cycle in response to stress signals induced by the accumulation of toxic forms of aSyn, perhaps as part of an aborted attempt to replace dying cells.

Our previously reported data (Sampaio‐Marques et al., 2012) and results presented here establish that autophagy, which is a fundamentally important pro‐survival mechanism, has deleterious effects on aged cells expressing aSyn. A pro‐death role for a survival pathway is apparently contradictory. However, although increased autophagic flux likely mediates aSyn clearance in functionally competent cells, it might also affect autophagy efficiency and selectivity in aged cells (Chu, 2011) that have a reduced ability to simultaneously upregulate anabolic processes.

**Figure 6:**
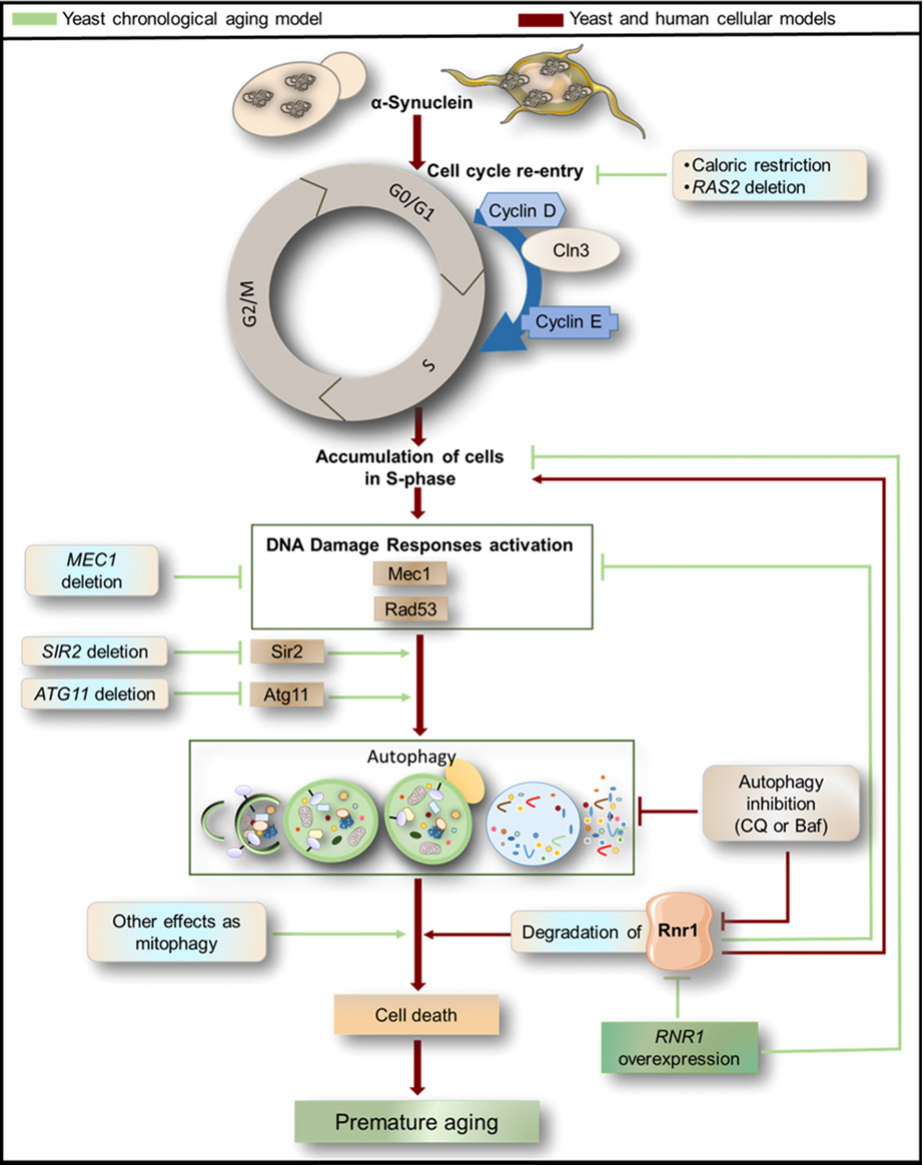
A model for aSyn toxicity that integrates inappropriate cell cycle re‐entry, activation of DNA damage responses, autophagy induction, and downregulation of ribonucleotide reductase 1 (Rnr1)

The results presented here indicate that the induction of DDR associated with the inappropriate entry into S‐phase of budding yeast cells in response to Ras2‐dependent growth signaling underlies the induction of autophagy that, in turn, leads to a reduction in levels of Rnr1 (Figure 6). This likely causes cells to remain trapped in S‐phase and triggers regulated cell death. Our data also demonstrate that cell death induced by expression of aSyn in budding yeast can be inhibited by overexpression of *RNR1* or by inhibition of DDR or autophagy. Thus, inappropriate re‐entry into S‐phase that induces DDR and autophagy upstream of the autophagic reduction in levels of Rnr1 is a proximate cause of aSyn toxicity in yeast (Figure 6). Interestingly, a connection between aSyn expression and regulation of Rnr1 levels in budding yeast was previously reported (Liu et al., 2011). However, in this earlier study, in contrast to the effects of toxic levels of aSyn expression in our study, low, nontoxic levels of aSyn increased, rather than decreased, levels of Rnr1. Together with our findings, these data point to a normal function of aSyn that involves upregulation of RNR to maintain genome stability, perhaps by promoting DNA replication and homologous recombinational repair of DNA damage when it occurs in quiescent cells, which is superseded by a switch to inhibition of RNR activity that induces cell death in cells expressing toxic levels of aSyn.

The proposed targeted mechanism of autophagy activation by DDR (Eapen et al., 2017) seems to be operational in the context of aSyn toxicity during chronological aging. In fact, we have previously shown that autophagy‐mediated aSyn‐induced premature aging is dependent on Atg11 (Sampaio‐Marques et al., 2012), which is also required for the genotoxin‐targeted autophagy (GTA) pathway (Eapen et al., 2017). Our previous data also demonstrated that autophagy induction and aSyn toxicity are dependent on sirtuin 2 (Sir2) (Sampaio‐Marques et al., 2012). Thus, a key question is as follows: How is autophagy dependent on DDR and/or Sir2? The role of sirtuins on genomic stability associated with the regulation of chromatin dynamics and protection of DNA from damage by modulation of DNA repair mechanisms is well known. Interestingly, aSyn has been shown to inhibit the activity of histone acetyltransferase (HAT) enzymes, thus deregulating the dynamic control of gene transcription (Kontopoulos, Parvin, & Feany, 2006). The relevance of sirtuins to neurodegeneration was recently highlighted in a report showing that SIRT2 modulates proteotoxicity associated with age‐related neurodegenerative disorders such as PD (de Oliveira et al., 2017). In yeast, in response to DNA damage, Sir2 is recruited by DDR via Mec1 and relocalizes to sites of DNA breakage (Mills, Sinclair, & Guarente, 1999). This suggests the interesting hypothesis that Sir2 might be involved together with DDR in the induction of the selective autophagy pathway in aSyn‐expressing cells. Our data demonstrating that forcing aSyn‐expressing cells to arrest in G0/G1 or abrogating DDR in these cells leads to maintenance of autophagy at homeostatic levels are consistent with this hypothesis.

In summary, our findings point to a model for the toxic effects of aSyn in synucleinopathies that involves the induction of RAS‐dependent growth signaling, which inappropriately causes postmitotic cells to re‐enter the cell cycle. This is followed by the activation of DDR that triggers autophagy and the inactivation of RNR. This likely causes a reduction in the dNTP pools, which traps cells in S‐phase and induces regulated cell death (Figure 6). These findings are remarkably similar to our earlier findings that elevated levels of growth signaling induced by high levels of glucose also lead to inappropriate re‐entry of quiescent, stationary‐phase budding yeast cells into the cell cycle, and regulated cell death, which is blocked by inactivating Ras2 or by overexpressing *RNR1* (Weinberger, Sampaio‐Marques, Ludovico, & Burhans, 2013). The similarities between these phenotypes and cancer suggest the intriguing possibility that some of the molecular events underlying synucleinopathies are caused by chronic hyperglycemia. Indeed, in addition to neoplastic disease (Weinberger et al., 2013), hyperglycemia and/or diabetes are risk factors for some neurodegenerative diseases including synucleinopathies (Ohara et al., 2011).

Multiple pathways of cellular dysfunction associated with aSyn aggregation have previously been implicated in models of synucleinopathies, including nuclear, mitochondrial, ER/Golgi, and autophagic dysfunction as well as dysfunctional organelle dynamics (Wong & Krainc, 2017). Although all of these dysfunctional pathways contribute to aSyn cytotoxicity, we consider it likely that many of these pathways converge on the conserved molecular events in postmitotic neurons related to dNTP metabolism that are predicted by our experiments. In this model, autophagy‐dependent reduction in levels of RNR activity and levels of dNTPs is the proximate cause of cell death of postmitotic neurons in many patients suffering from synucleinopathies, downstream of cellular dysfunction associated with aSyn aggregation. This provides the opportunity to develop more effective therapeutic strategies for treating these patients by targeting events that directly impact growth signaling and/or dNTP metabolism.

## 4 EXPERIMENTAL PROCEDURES

### 4.1 Yeast strains and plasmids

The yeast strains and plasmids used in this study are listed in Tables 1 and 2.

**Table 1:** Yeast strains used in this study

Yeast strain	Genotype
BY4742	*MATα his3Δ1 leu2Δ0 lys2Δ0 ura3Δ0*
W303‐1A	*MATa ade2‐1 trp1‐1 ura3‐1 leu2‐3,112 his 3‐11,15 can1‐100* *rad5‐535*
*ras2Δ*	BY4742 *ras2::kanMX4*
*cln3Δ*	BY4742 *cln3::kanMX4*
*rim15Δ*	BY4742 *rim15::kanMX4*
*ydj1Δ*	BY4742 *ydj1::kanMX4*
U953‐61A	W303‐1A *mec1::TRP1 sml1::HIS3*
U952‐3B	W303‐1A *sml1::HIS3*

**Table 2:** Plasmids used in this study

Plasmids	Type of plasmid	Source
pYX222	2 µ	Sampaio‐Marques et al. (2012)
pYX222‐wt_aSyn	2 µ	Sampaio‐Marques et al. (2012)
pYX222‐A30P_aSyn	2 µ	Sampaio‐Marques et al. (2012)
pYX242	2 µ	Sampaio‐Marques et al. (2012)
pYX242‐wt_aSyn	2 µ	Sampaio‐Marques et al. (2012)
pYX242‐ A30P_aSyn	2 µ	Sampaio‐Marques et al. (2012)
YEp24	2 µ	Weinberger et al. (2007)
YEp24_RNR1	2 µ	Kaeberlein, Burtner, and Kennedy (2007)
pRS416‐GFPAtg8	2 µ	Guedes et al. (2016)
pcDNA3.1	2 µ	Zondler et al. (2014)
pcDNA3.1wt_aSyn	2 µ	Zondler et al. (2014)

### 4.2 Cell growth and culture conditions and measurement of cell survival

Yeast cells were cultured in selective YNB medium (Difco Laboratories) supplemented with 100 g/L uracil, 300 mg/L leucine, 50 g/L histidine, 50 g/L lysine, 100 g/L tryptophan, and 100 g/L adenine. Cultures were grown overnight at 26°C, 150 rpm. Survival was assessed by colony‐forming units (CFUs) after 2 days of incubation at 30ºC on YEPD (0.5% yeast extract, 1% peptone, 2% glucose, and 2% agar) agar plates.

Human H4 neuroglioma cells were maintained in Opti‐MEM medium (Thermo Fisher) supplemented with 10% fetal bovine serum and incubated at 37°C, 5% CO_2_. Viability was assessed using the CellTiter 96 AQueous One Solution Cell Proliferation Assay (MTS) (Promega) according to the manufacturer's instructions.

### 4.3 Chronological lifespan assays

Cells were grown on synthetic liquid media until reaching stationary phase, and this time point was considered day 0 of chronological lifespan (CLS). Samples for survival assessment were collected from day 0 of CLS (when viability was considered to be 100%) and then again every 2–3 days until less than 0.01% of the cells in the culture were viable. Mean (50% survival) lifespan and maximum (10% survival) lifespan were determined from curve fitting of the survival data (from pair‐matched, pooled experiments) with the statistical software Prism (GraphPad Software).

### 4.4 H4 cell transient transfection

H4 cells were plated at 150,000 cells/ml 12 hr prior to transfection. Cells were transfected with FuGENE 6 Transfection Reagent (Promega) according to the manufacturer's instructions with the vector control or the pcDNA3.1 vector encoding aSyn and 24 hr later assayed.

### 4.5 Cell cycle analysis

Cell cycle analysis in yeast cells was performed essentially as previously described (Fortuna et al., 2001). A BD LSR II (Becton Dickinson, NJ, USA) with a 488‐nm excitation laser was used. Signals from 30,000 cells/sample were captured in FITC channel (530 nm ± 30 nm), at a flow rate of about 500 cells/s.

H4 cells were collected, and a pool of adherent and suspended cells was made. Cells were then pelleted, washed, and fixed with ethanol (70% v/v) for at least 30 min at 4ºC. After fixation, cells were rinsed with PBS and incubated with staining solution (PBS with 0.1% (v/v) Triton X‐100; 20 µg/ml of propidium iodide (PI) (Molecular Probes); 250 µg/ml of RNase) for one hour at 50°C, in the dark. A BD LSR II with a 488‐nm excitation laser was used. Signals from 15,000 cells/sample were captured in a PI channel (585 nm ± 40 nm), at a flow rate of about 500 cells/s.

Cell cycle profile was determined by the percentage of cells in each phase of the cell cycle (ModFit LT software v3.2; Verity Software House, Topsham, ME, USA).

### 4.6 Budding index

The budding status of at least 300 individual yeast cells from each sample was visually determined using an Olympus BX60 microscope. Cell clumps were dissociated by sonication (Vibra‐Cell™ Ultrasonic Liquid Processor—VC 130).

### 4.7 Preparation of protein extracts

Yeast cells were disrupted using glass beads in cold lysis buffer (1%v/v Triton X‐100, 120 mM NaCl, 50 mM Tris‐HCl pH 7.4, 2 mM EDTA, 10%v/v glycerol, 1 mM PMSF, and Complete Mini Protease Inhibitor Cocktail) on a FastPrep‐24 Classic Instrument (MP Biomedicals) during 5 cycles of 45‐s beating and 3‐min pause. Protein extracts were then collected by centrifugation at 10,000 *g* during 30 min.

Protein extraction for Rad53 detection involved the pretreatment of the cells with 2 M lithium acetate for 5 min at room temperature. After lithium acetate removal, 0.4 M NaOH was added for 5 min on ice, and then, the protocol for protein extraction was followed.

For H4 cells, protein extraction was performed with lysis buffer (1% NP‐40, 500 mM Tris‐HCL, 2.5 M NaCl, 20 mM EDTA, and phosphatase and protease inhibitors, at pH 7.2). Samples were incubated for 30 min at 4°C with agitation, sonicated in an ultrasonic ice‐cold bath for 15 min, and centrifuged at 13,000 rpm during 30 min; the supernatant was considered the total protein extract.

### 4.8 Western blot

Western blots of protein extracts from both yeast and H4 cells were carried out by resolving 20 μg of the total protein on a 12% SDS–polyacrylamide gel and transferred to a nitrocellulose membrane for 7–15 min in Trans‐Blot Turbo Transfer System (Bio‐Rad). For Rad53 detection, extracts were overrun in a 7.5% SDS–polyacrylamide gel. As a positive control for DDR activation, extracts from cells treated with 50 mM of hydroxyurea (HU) for 6 hr were used. Membranes were immunoblotted with the primary antibodies. Rabbit anti‐α‐synuclein (1:1,000; Sigma Aldrich), goat anti‐GFP (1:5,000; Abcam), rabbit anti‐Rad53 (1:5,000; Abcam), goat anti‐Cln3 (1:500; Santa Cruz), mouse anti‐Pgk1 (1:5,000; Invitrogen), mouse anti‐GAPDH (1:200; Millipore), rabbit anti‐Rnr1 (1:5,000; Agrisera), rabbit anti‐Rnr3 (1:1,000; Agrisera), rabbit anti‐cyclin D1 (1:1,000; Cell Signaling), mouse anti‐cyclin E (1:500; Invitrogen), goat anti‐actin (1:5,000; from C. Gourlay laboratory), rabbit anti‐LC3A/B (1:1,000; Cell Signaling), mouse anti‐p62 (1:1,000; Abcam), and mouse anti‐alpha‐actin (1:1,000; Millipore) were used. Secondary antibodies (HRP, anti‐rabbit, anti‐mouse, and anti‐goat) were from Bio‐Rad (1:5,000). Blots were treated with the SuperSignal West Femto Maximum Sensitivity Substrate (Thermo Fisher) or Clarity Western ECL Substrate (Bio‐Rad). Digital images of the Western blots were obtained in a ChemiDoc XRS System (Bio‐Rad) with Quantity One software V4.6.5 (Bio‐Rad). Immunoblot bands were quantified by Quantity One software.

### 4.9 Monitoring autophagy by GFP‐Atg8 assay

The delivery of Atg8 to the vacuole was followed by the GFP N‐terminally tagged to Atg8 (Cheong & Klionsky, 2008). As GFP is more resistant to the vacuolar degradation than Atg8, bulk autophagy results in the accumulation of free GFP in the vacuole. GFP‐Atg8 and free GFP are detectable by Western blotting using a GFP‐specific antibody. To perform this assay, cells were transformed with the plasmid pRS416‐GFPAtg8 with fusion gene under the control of the *ATG8* endogenous promoter. The ratio between free GFP and total GFP is used as a readout for the autophagic flux. Immunoblot bands were quantified by densitometric analysis using the Quantity One software.

### 4.10 Immunofluorescence staining

H4 cells were resuspended in PBS, 24 hr after transfection, and were fixed with 2% PFA during 15 min at RT. Cells were then washed, permeabilized, and blocked with 4% BSA in PBS 0.05% Tween. Primary antibody, anti‐LC3 A/B (1:250), was incubated overnight at 4°C. Anti‐Rabbit IgG Alexa Fluor 488 green fluorescent dye (Molecular Probes) was used as secondary antibody. Nuclei were stained with DAPI (4’,6‐diamidino‐2‐phenylindole). An Epifluorescence Microscope (BX61 microscope with an Olympus DP70 camera) was used, and images were analyzed with ImageJ^®^ software v1.49 (NIH).

### 4.11 Quantitative mRNA expression

mRNA expression analysis was performed as described (Sampaio‐Marques et al., 2012). Quantitative real‐time PCR (qPCR) was used to measure the mRNA transcripts of the *CLN3* (F: 5’TGAGCATCCCACAAAATTCA3’; R: 5’AGTGGCCATGGGTCTAACAG3’), *CLN1 *(F: 5’CAACATTGACCATTCATCGCC3’; R: 5’GGGTTTGATTAGGTAGACTGC3’), *CLN2 *(F: 5’ CCGTTAGTGTGAATAGTCTGG3’; R: 5’CGTTGCGGGATTTTGGTTTTC3’), *CLB5 *(F: 5’GCCCAACCCACTCAATTTCCTAAG3’; R: 5’GAATGAATTGGTGGCAGCAGTAGG3’), *CLB6 *(F: 5’ATCACTTGCCTGTTCATTGCC3’; R: 5’CAGCCTTCCTAATTCCTTCGAC3’), and *RNR3 *(F: 5’CAGAACGTCCTCAGCATTTG3’; R: 5’GTACCAGCGATATAAGAACC3’) genes.

### 4.12 Quantification and statistical analysis

Quantification analysis and statistical tests performed are described in the corresponding figure legends. Statistical analysis was performed by two‐way ANOVA in the yeast cell assays and by parametric two‐tailed Student's *t* test (normal distribution assumed) in the H4 cell assays. A p‐value of less than 0.05 was considered a significant difference. For the statistical analysis, GraphPad Prism software v5.01 was used. Data presented in figures refer to mean ± *SEM* of at least three independent biological replicates.

## CONFLICT OF INTEREST

None Declared.

## AUTHOR CONTRIBUTIONS

PL, WCB, JW, BSM, and TFO conceived and designed the experiments; BSM, AG, IV, and SAG acquired the data; PL, BSM, WCB, JW, and TFO analyzed and interpreted the data; PL, BSM, and WCB with input from other authors wrote the paper. All authors approved the final manuscript.
