# Supplementary material for: α‐Synuclein toxicity in yeast and human cells is caused by cell cycle re‐entry and autophagy degradation of ribonucleotide reductase 1
Source: Aging Cell. 2019 Apr 11;18(4):e12922. doi: 10.1111/acel.12922 (PMC6612645; doi:10.1111/acel.12922)
Supplement: Supplementary file 1 [file ACEL-18-e12922-s001.docx]

**
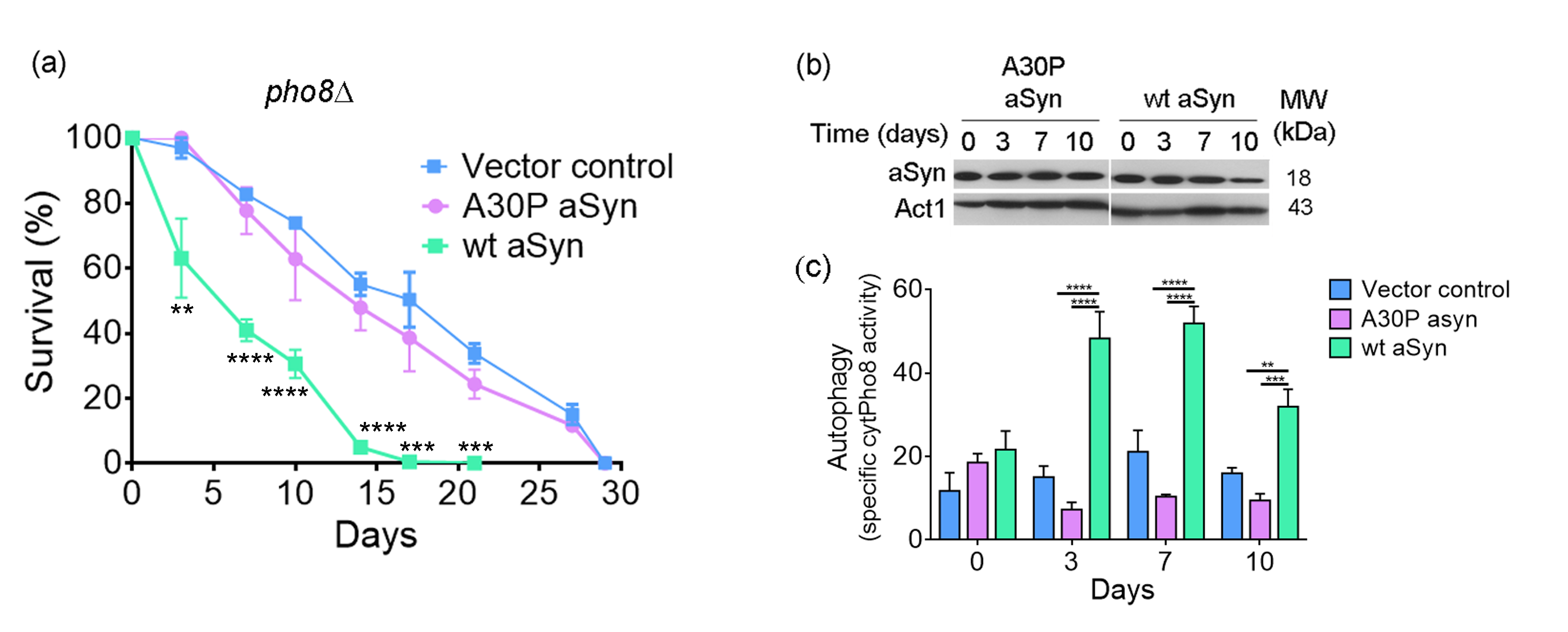
**

**Supplementary Figure S1.** **α-Synuclein (aSyn) induces premature aging accompanied by increased autophagy as revealed by the quantitative Pho8Delta60 assay.** (a) Chronological lifespan (CLS) and (b) aSyn levels of *pho8Δ* cells expressing the vector control, wt aSyn or A30P aSyn variant. (c) Autophagy activity of *pho8Δ* cells expressing the vector control, wt aSyn or A30P aSyn variant determined by the alkaline phosphatase assay (ALP) ([Sampaio-Marques et al., 2012](#_ENREF_34)). To assess autophagy, *PHO8* mutant cells were transformed with a plasmid expressing an inactive Pho8 proenzyme target to the cytosol, to assess autophagy ([Sampaio-Marques et al., 2012](#_ENREF_34)). Significance of the data was determined by two-way ANOVA (**p≤0.01; ***p≤0.001, ****p≤0.0001) comparing *pho8Δ* cells expressing vector control or the aSyn variants.
