# Supplementary material for: α‐Synuclein toxicity in yeast and human cells is caused by cell cycle re‐entry and autophagy degradation of ribonucleotide reductase 1
Source: Aging Cell. 2019 Apr 11;18(4):e12922. doi: 10.1111/acel.12922 (PMC6612645; doi:10.1111/acel.12922)
Supplement: Supplementary file 2 [file ACEL-18-e12922-s002.docx]

**
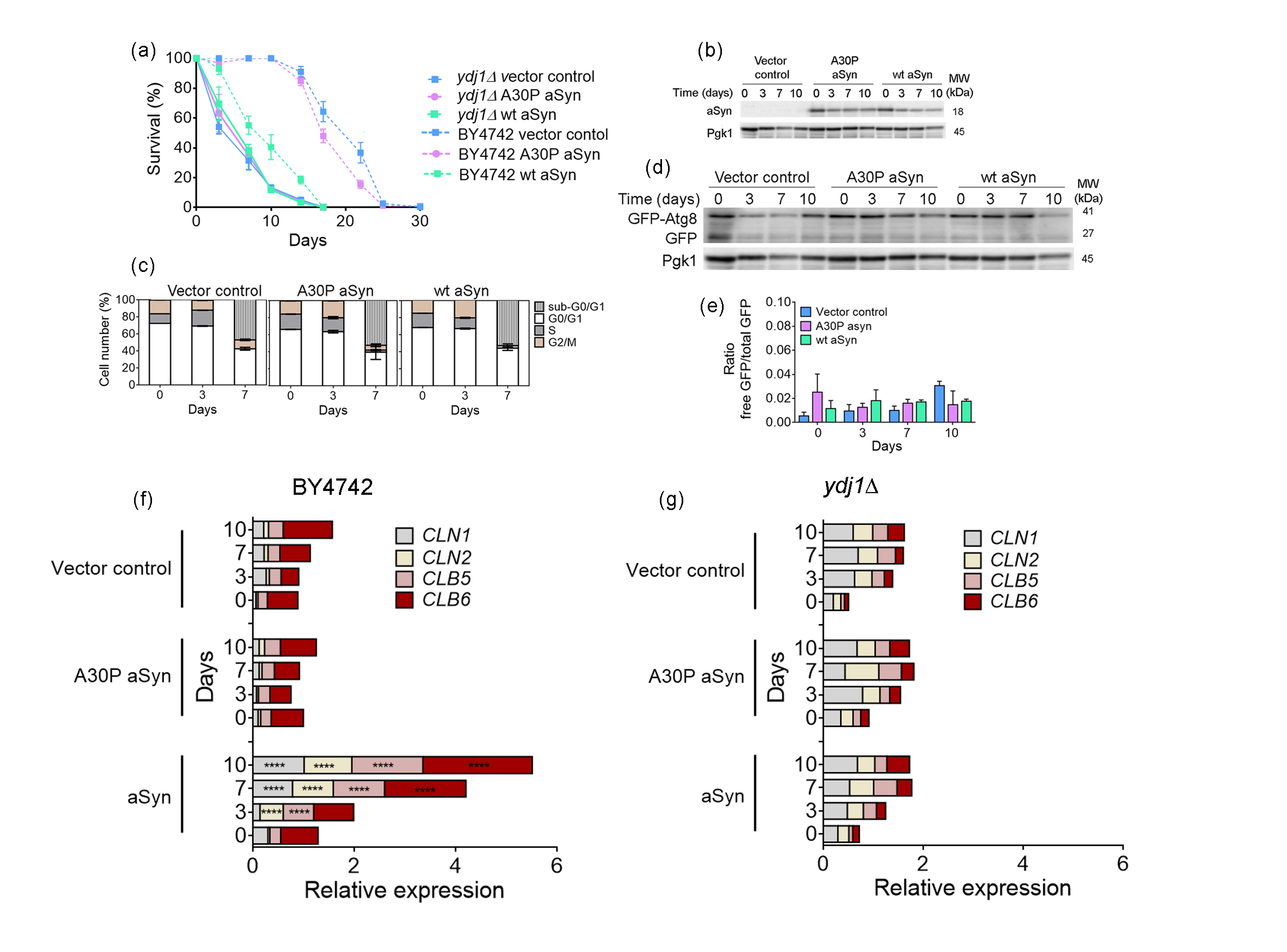
**

**Supplementary Figure S2.** **aSyn toxicity is dependent of the molecular chaperon Ydj1**. (a) Chronological lifespan (CLS) and (b) aSyn levels of *ydj1Δ* cells expressing the vector control, wt aSyn or A30P aSyn variant (CLS of BY4742, dashed lines, are repeated from figure 1a to facilitate interpretation). (c) Cell cycle measurements of DNA content by flow cytometry. (d) Representative blot of GFP detection for the evaluation of autophagy by the GFP-Atg8 processing assay. (e) Graphical representation of the ratio between the free GFP versus the total GFP obtained by densitometric analysis of the gels used on the evaluation of autophagy by the GFP-Atg8 processing assay. Relative *CLN1, CLN2, CLB5 and CLB6* mRNA expression levels of BY4742 (f) and *ydj1Δ* (g) cells expressing the vector control or aSyn variants. Three reference genes (*ACT1*-actin*, PDA1*-alpha subunit of pyruvate dehydrogenase and *TDH2*-isoform 2 of glyceraldehyde-3-phosphate dehydrogenase) were used as internal standards for the normalization of mRNA expression levels. Immunoblot bands were quantified by Quantity One software. Significance of the data was determined by two-way ANOVA (****p≤0.0001) comparing BY4742 cells expressing aSyn with cells expressing the vector control or the A30P aSyn variant. The same analysis was performed for *ydj1Δ* cells. Data represent mean ± SEM of three independent biological replicates.
