# Supplementary material for: α‐Synuclein toxicity in yeast and human cells is caused by cell cycle re‐entry and autophagy degradation of ribonucleotide reductase 1
Source: Aging Cell. 2019 Apr 11;18(4):e12922. doi: 10.1111/acel.12922 (PMC6612645; doi:10.1111/acel.12922)
Supplement: Supplementary file 3 [file ACEL-18-e12922-s003.docx]

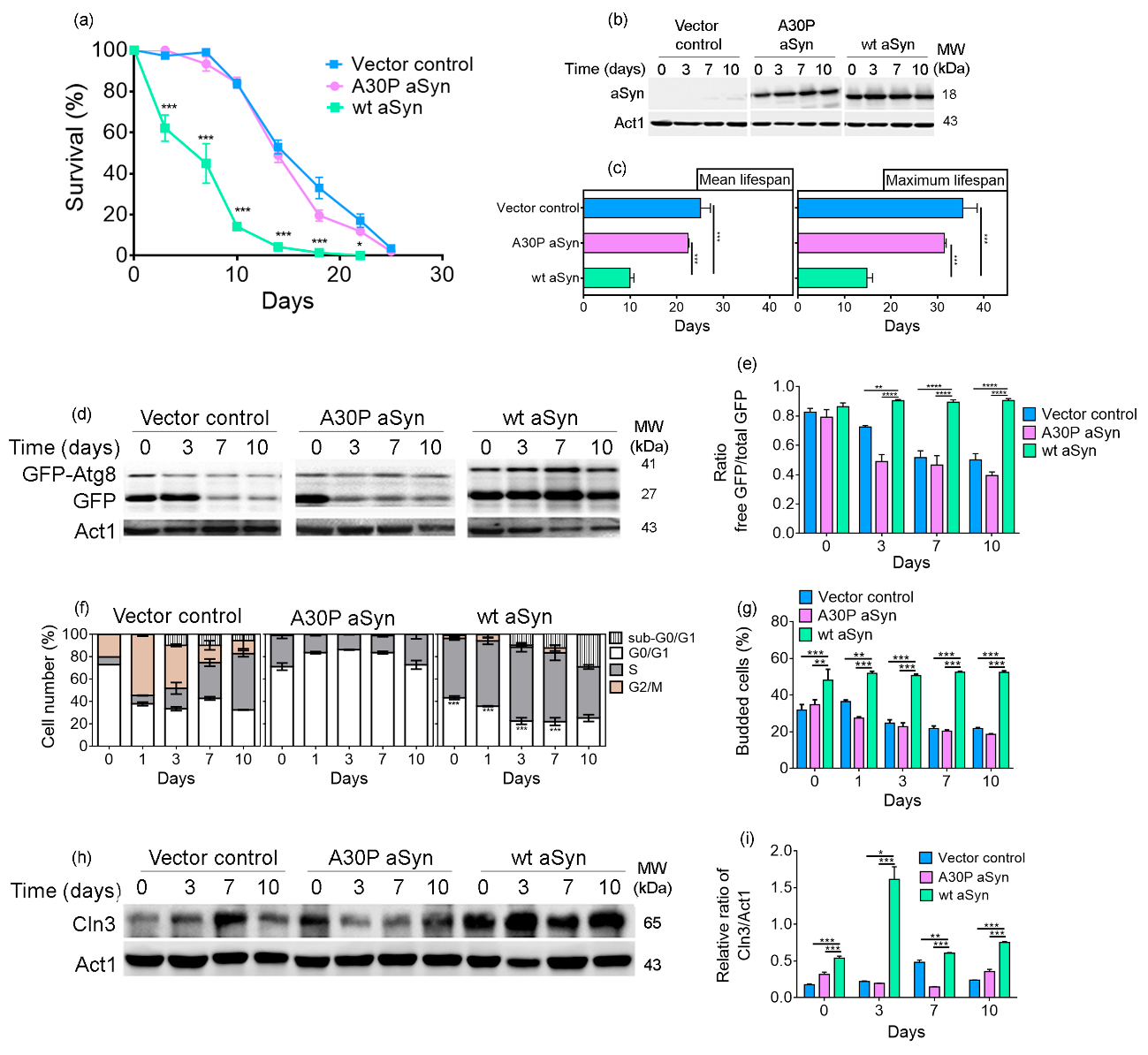


**Supplementary Figure S3.** **α-Synuclein (aSyn) promotes cell cycle re-entry and S-phase arrest associated with increased autophagy in W303-1A cells.** (a) Chronological lifespan (CLS) and (b) aSyn levels of W303-1A cells expressing the vector control, wt aSyn or A30P aSyn variant. (c) Mean (50% survival) and maximum (10% survival) CLS were determined from curve fitting of the survival data (from pair-matched, pooled experiments) with the statistical software Prism (GraphPad Software). (d) Representative blot of GFP detection for the evaluation of autophagy by the GFP-Atg8 processing assay. (e) Graphical representation of the ratio between the free GFP versus the total GFP obtained by densitometric analysis of the gels used on the evaluation of autophagy by the GFP-Atg8 processing assay. (f) Cell cycle measurements of DNA content by flow cytometry. (g) Bud index indicating the percentage of cells with visible buds. (h) Representative blot of Cln3 immunoprecipitation and detection by anti-Cln3 antibody. (i) Graphical representation of the ratio between Cln3 and Act1 obtained by densitometric analysis. Immunoblot bands were quantified by Quantity One software. Significance of the data was determined by two-way ANOVA (*p≤0.05, **p≤0.01; ***p≤0.001, ****p≤0.0001) comparing wild type cells expressing vector control or the aSyn variants. Data represent mean ± SEM of three independent biological replicates.
