# Supplementary material for: α‐Synuclein toxicity in yeast and human cells is caused by cell cycle re‐entry and autophagy degradation of ribonucleotide reductase 1
Source: Aging Cell. 2019 Apr 11;18(4):e12922. doi: 10.1111/acel.12922 (PMC6612645; doi:10.1111/acel.12922)
Supplement: Supplementary file 4 [file ACEL-18-e12922-s004.docx]

**
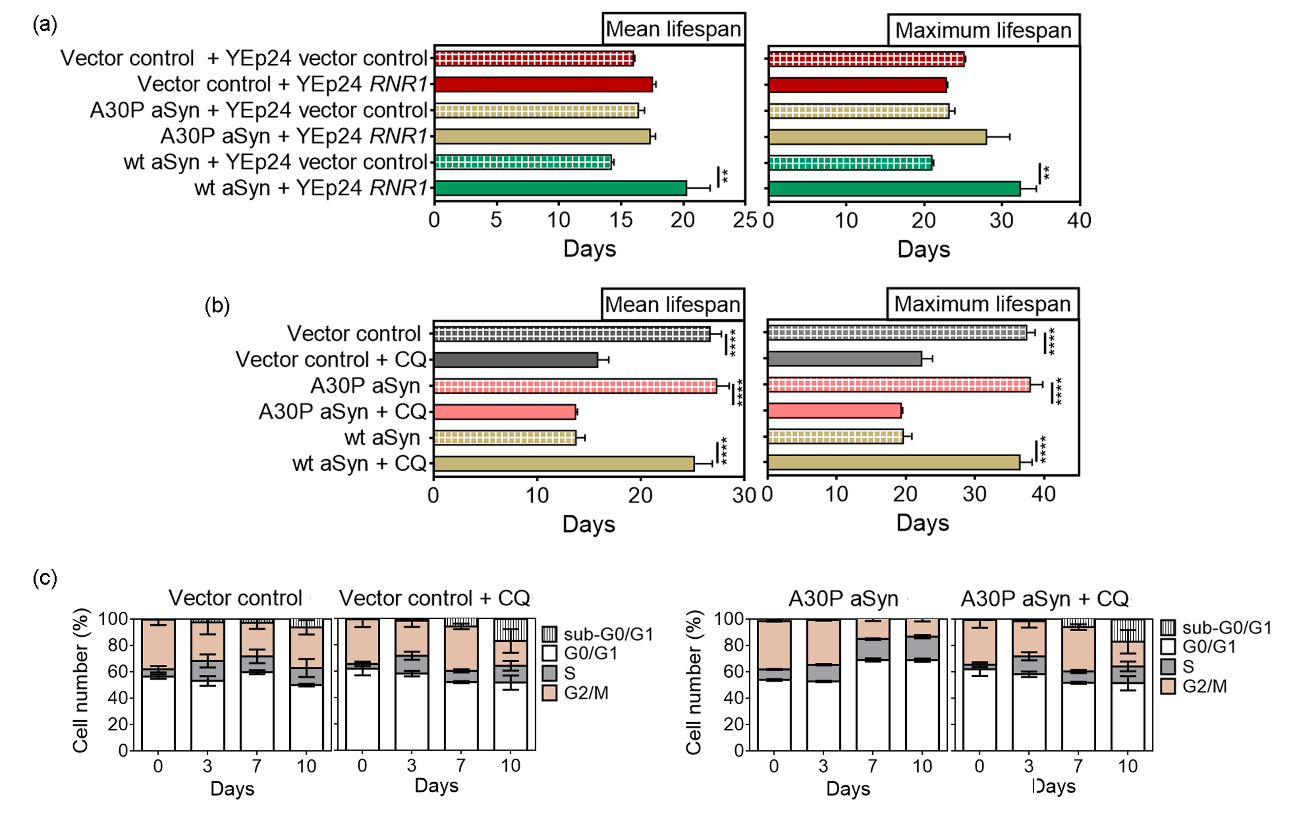
**

**Supplementary Figure S4.** ***RNR1* overexpression or autophagy inhibition ameliorates α-Synuclein (aSyn)-mediated toxicity.** (a) Mean (50% survival) and maximum (10% survival) lifespans of BY4742 cells co-expressing the vector control, wt aSyn or A30P aSyn variant and *RNR1* or the vector control. Mean and maximum lifespans were determined from curve fitting of the survival data (Figure 4c) from pair matched, pooled experiments with the statistical software Prism (GraphPad Software). Significance of the data was determined by two-way ANOVA (**p≤0.01) comparing cells co-expressing *RNR1* and the vector control or the aSyn variants. (b) Mean and maximum lifespans of BY4742 cells expressing the vector control, wt aSyn or A30P aSyn variant in the presence or absence of 50 µg/µl chloroquine (CQ) (autophagy inhibitor). Mean and maximum lifespans were determined from curve fitting of the survival data (Figure 4i) from pair matched, pooled experiments with the statistical software Prism (GraphPad Software). (c) Cell cycle measurements of DNA content by flow cytometry of BY4742 cells expressing the vector control or A30P aSyn variant treated or not with CQ. (Data relatively to BY4742 cells expressing the vector control or the A30P aSyn variant, without CQ treatment are repeated from Figure 1 to facilitate their interpretation). Significance of the data presented in (b) and (c) was determined by two-way ANOVA (****p≤0.0001) comparing BY4742 cells expressing the vector control, wt aSyn or A30P aSyn variant cells in the presence or absence of CQ.
